# Land‐use intensity and the effects of organic farming on biodiversity: a hierarchical meta‐analysis

**DOI:** 10.1111/1365-2664.12219

**Published:** 2014-02-07

**Authors:** Sean L. Tuck, Camilla Winqvist, Flávia Mota, Johan Ahnström, Lindsay A. Turnbull, Janne Bengtsson

**Affiliations:** ^1^ Department of Plant Sciences University of Oxford Oxford OX1 3RB UK; ^2^ Section for Landscape and Soil Ecology Department of Ecology SLU Box 7044 Uppsala S‐750 07 Sweden; ^3^ Institute of Evolutionary Biology and Environmental Studies University of Zurich Zurich 8057 Switzerland

**Keywords:** agricultural management, diversity, farming systems, landscape complexity, species richness

## Abstract

1. The benefits of organic farming to biodiversity in agricultural landscapes continue to be hotly debated, emphasizing the importance of precisely quantifying the effect of organic vs. conventional farming.
2. We conducted an updated hierarchical meta‐analysis of studies that compared biodiversity under organic and conventional farming methods, measured as species richness. We calculated effect sizes for 184 observations garnered from 94 studies, and for each study, we obtained three standardized measures reflecting land‐use intensity. We investigated the stability of effect sizes through time, publication bias due to the ‘file drawer’ problem, and consider whether the current literature is representative of global organic farming patterns.
3. On average, organic farming increased species richness by about 30%. This result has been robust over the last 30 years of published studies and shows no sign of diminishing.
4. Organic farming had a greater effect on biodiversity as the percentage of the landscape consisting of arable fields increased, that is, it is higher in intensively farmed regions. The average effect size and the response to agricultural intensification depend on taxonomic group, functional group and crop type.
5. There is some evidence for publication bias in the literature; however, our results are robust to its impact. Current studies are heavily biased towards northern and western Europe and North America, while other regions with large areas of organic farming remain poorly investigated.
6. *Synthesis and applications*. Our analysis affirms that organic farming has large positive effects on biodiversity compared with conventional farming, but that the effect size varies with the organism group and crop studied, and is greater in landscapes with higher land‐use intensity. Decisions about where to site organic farms to maximize biodiversity will, however, depend on the costs as well as the potential benefits. Current studies have been heavily biased towards agricultural systems in the developed world. We recommend that future studies pay greater attention to other regions, in particular, areas with tropical, subtropical and Mediterranean climates, in which very few studies have been conducted.

## Abstract 2

Our analysis affirms that organic farming has large positive effects on biodiversity compared with conventional farming, but that the effect size varies with the organism group and crop studied, and is greater in landscapes with higher land‐use intensity. Decisions about where to site organic farms to maximize biodiversity will, however, depend on the costs as well as the potential benefits. Current studies have been heavily biased towards agricultural systems in the developed world. We recommend that future studies pay greater attention to other regions, in particular, areas with tropical, subtropical and Mediterranean climates, in which very few studies have been conducted.

## Introduction

Organic farming, in which insecticides, herbicides and inorganic fertilizers are entirely or largely avoided, is generally thought to be more environmentally benign than its conventional farming cousin. However, the overall benefits of organic farming for biodiversity, the environment in general, human health and food security have been intensely debated in recent years (Bengtsson, Ahnström & Weibull 2005; Hole *et al*. 2005; Badgley *et al*. 2007; Mondelaers, Aertsens & Huylenbroeck 2009; Dobermann 2012; Reganold 2012; Tuomisto *et al*. 2012; Winqvist, Ahnström & Bengtsson 2012; Gabriel *et al*. 2013). The debate turns on whether or not the decreased yields from organic farms negate any local benefits, for example, to biodiversity, that such methods deliver (Seufert, Ramankutty & Foley 2012; but see Badgley *et al*. 2007). The logic of this argument runs as follows: lower yields push up food prices, and as a consequence, more wild or marginal land is brought into agricultural production. This wild land is likely to have supported even higher biodiversity than the organic farm; hence, begging the question, is there an overall cost of organic farming to biodiversity?

Organic farming provides shared benefits to both humans and wildlife, while conventional farming, at least in the short term, maximizes yields – thus potentially sparing wild lands elsewhere – therefore this argument is often naively framed as ‘land sharing’ vs. ‘land sparing’ (Green *et al*. 2005; Vandermeer & Perfecto 2007; Fischer *et al*. 2008; Phalan *et al*. 2011; Tscharntke *et al*. 2012; Gabriel *et al*. 2013) although recently the debate has moved away from such overly simplistic dichotomies. For example, it has been argued that decisions about land sparing vs. sharing are contingent on the landscape and potential yields (Hodgson *et al*. 2010; Tscharntke *et al*. 2012; Gabriel *et al*. 2013). It is also clear that some organisms are necessary on the farm to support essential ecosystem services, for example, pollination and pest control, which contribute to yield. Therefore, species in farmland cannot be entirely sacrificed in order to preserve biodiversity elsewhere. In addition, some species, particularly in Europe where farming has been an integral part of the landscape for thousands of years, thrive in extensively managed farmland and are clearly threatened by agricultural intensification (Chamberlain *et al*. 2000). These species are an integral part of the European cultural landscape, and their loss has provoked both public and political outcry, leading the British Government, for example, to pledge to reverse such declines by 2020. Thus, organic farming, which generally increases both crop and landscape heterogeneity, may be one component of a land‐sharing strategy, delivering wider ecosystem services including amenity and conservation of culturally important species (Vandermeer & Perfecto 2007; Gabriel *et al*. 2013). In this light, quantifying the precise benefits delivered by organic farming is essential.

While there is a general consensus that organic farming increases biodiversity when compared to conventional agriculture, the magnitude of this effect seems to vary greatly, particularly among organism groups and across landscapes (Bengtsson, Ahnström & Weibull 2005; Batáry *et al*. 2011; Winqvist, Ahnström & Bengtsson 2012). Bengtsson, Ahnström and Weibull (2005) suggested that the effects of organic farming on biodiversity were likely to be greatest in intensively managed agricultural landscapes, while Tscharntke *et al*. (2005) argued that agrienvironment schemes would have larger effects in simple than in complex landscapes. Some of these predictions have been borne out by individual studies (Rundlöf & Smith 2006; Rundlöf, Bengtsson & Smith 2008; Brittain *et al*. 2010; Diekötter *et al*. 2010; Batáry *et al*. 2011; Fischer *et al*. 2011; Flohre *et al*. 2011; Winqvist, Ahnström & Bengtsson 2012) and by meta‐analysis in which landscapes were classified as either simple or complex (Batáry *et al*. 2011). However, different studies have defined ‘simple’ and ‘complex’ in different ways, whereas it would be preferable to have some more objective, continuous measurement of land‐use intensity with which to test these ideas more fully.

While there have been previous meta‐analyses comparing conventional vs. organic farming and their biodiversity and environmental impacts (Bengtsson, Ahnström & Weibull 2005; Batáry *et al*. 2011; Seufert, Ramankutty & Foley 2012; Tuomisto *et al*. 2012), we believe that a new analysis is still timely. First, previous meta‐analyses have not taken account of the hierarchical structure of the data; secondly, a large number of new studies have been published in recent years; and thirdly, we include here three objective and standardized measures of land‐use intensity and landscape complexity measured on a continuous scale, newly obtained for each of the studies. Using an extended data set compared with Bengtsson, Ahnström and Weibull (2005), we can therefore ask the following questions: (i) By how much does organic farming increase biodiversity compared with conventional agriculture? (ii) Do the effects of organic farming depend on the organism or functional group, land‐use intensity and structure, and crop type? (iii) Has the reported effect size of organic farming on biodiversity decreased or remained stable over time? (iv) Is there evidence for publication bias in the literature, either because studies with negligible or negative effects of organic farming remain unpublished or because the present studies of organic farming, which are often performed in Europe or the US (Batáry *et al*. 2011; Winqvist, Ahnström & Bengtsson 2012), are unrepresentative of the crops and regions in which organic farming is conducted globally?

## Materials and methods

### Data Collection

We started with the species richness data set published in 2005 by Bengtsson, Ahnström and Weibull, which included 27 studies published before December 2002. We expanded this data set to include an additional 68 studies published between 2003 and 2011. Some of the additional data (2003–2009) were gathered for an unpublished Master's thesis (Mota 2010). Further studies from 2010 to 2011 were added by co‐authors Ahnström and Winqvist, finishing the literature search by the end of 2011. The full data set consists of 94 publications (see Appendix S1 in Supporting information). When updating the data set of Bengtsson, Ahnström and Weibull (2005), we used the same keywords in ISI web of knowledge: biodiversity, biological diversity, conventional farming (agriculture) and organic farming (agriculture). We searched for additional studies by scanning the bibliographies in publications identified from our search. We followed the relevant literature and discussed with colleagues throughout. Our data set contains results from technical reports as well as peer‐reviewed journals. Although it is unlikely that the data we present are complete, we believe these studies are an extensive and representative sample.

For a publication to be included in the analysis, it had to provide species richness data (*n *>* *1) in both organic and conventional systems. This could be in the form of raw data or the mean species richness, standard deviation and sample size in both farming systems. In some cases, we used other richness data provided in the publications – for example, Shannon H’ (Mäder *et al*. 2002; Martínez‐Sánchez 2008) or richness of taxa higher than species level (e.g. Galván *et al*. 2009; Crowder *et al*. 2010). Unfortunately, many of the published studies do not meet these criteria and therefore did not provide sufficient data to be useful in a meta‐analysis (see Appendix S2, Table S3 Supporting information).

Organic agriculture is normally defined as any farming system where the use of pesticides, herbicides and synthetic fertilizers is prohibited or strictly limited. Organic farms often have other differences, for example they tend to use more complex crop rotations as a weed‐ and pest‐control strategy and use animal manure, green manure or compost in place of synthetic fertilizers. Conventional systems, however, use pesticides and inorganic fertilizers to various degrees and often use simplified crop rotations and fewer crops. Due to the broad range of farming systems that can be grouped within organic and conventional definitions, the two farming systems are likely to differ between and within studies. However, despite these potential differences, we did not further subdivide farming systems to avoid using more than two treatments in the meta‐analysis.

For each effect size, we extracted taxonomic and functional data on the study organism(s). We also recorded (i) the sampling unit of the species richness data (e.g. numbers per trap or transect), (ii) the sampling scale (plot, field or farm) and (iii) the crop type. Data on species richness were extracted from the text, tables or figures in publications using the program getdata graph digitizer 2.25 (Fedorov 2013) when necessary. Other measures of variation presented in publications were converted to standard deviations.

The information on taxonomic groups was used to create categorical covariates for different higher taxonomic units and ecological functions. For taxonomic groups, we classified species as: arthropods, birds, microbes and plants. Data on earthworms, mammals, nematodes and protozoa were excluded from this analysis due to small sample sizes (*n *<* *5). For functional groups, we classified species as producers (plants), herbivores, pollinators (as adults), predators, soil‐living decomposers and others (including omnivores and organisms with variable or unknown functional characteristics). The functional classification is based on the idea that different organism groups may contribute to different ecosystem services. We acknowledge that considerable uncertainty about ecological function exists for several groups: carabid beetles, for example, are often considered to provide pest control (Östman, Ekbom & Bengtsson 2001, 2003), but many species are known to be at least partly herbivorous seed eaters (Jonason *et al*. 2013).

We also separated the data according to crop type. Given the data, we were able to identify the following crop types: cereals, grassland (usually permanent or semi‐permanent leys or pastures), mixed crops (comparison made across several different crops), orchards, vegetable crops and miscellaneous (i.e. not specified precisely in the original study). Many studies include multiple records for different organism groups or crop types on the same farm. These were treated as distinct within‐study observations and used to calculate separate effect sizes for subgroups. As a result, our data set of 94 studies was subdivided into 184 observations (see Statistical Analysis for more details).

### Land‐Use Intensity Metrics

Three metrics of land‐use intensity were collected using Google Earth (2013). We conducted new landscape analyses for all included studies in order to provide continuous standardized measures of land‐use intensity and complexity. We distinguished between different land‐use types: field (annual and perennial crops, ley, grazed ley), pasture (perennial grassland used for grazing), forest (including clear‐cuts), wetland, water, rural, urban and permanent line elements (e.g. ditches, hedge rows, roads etc.). Using these land cover classifications, we calculated (i) % arable fields – the proportion of the landscape covered by arable fields; (ii) number of habitats – the number of distinguishable habitats found in the landscape; and (iii) average field size – the average size of arable fields in the landscape. The percentage of arable fields is a measure of land‐use intensity, while the number of habitats represents landscape complexity. However, an intensively farmed region is likely to include fewer habitats than a more extensively farmed area. The average field size may reflect the overall extent of farming on the landscape but, depending on local farming practices, not necessarily farming intensity.

To calculate the three metrics, we first identified a standardized sampling space at each location based on descriptions in the original publications. Where coordinates were not provided, we identified an area that we were confident, included the study area based on descriptions in the text. We then identified a central measuring point, making sure it was placed in a landscape with agricultural fields, and the radius (in metres) defining the appropriate area for sampling around this point. If no information about the area of the study region was available, we visually examined the Google Map image and set the radius so that the included landscape was representative of its complexity (and similar to the landscape closest to the central point). We then randomly placed five 1‐km transects within this study region. The positions of the five transects were defined by sets of three randomly generated numbers. The first number, randomly selected between 0 (central measuring point) and the radius of the study region, denoted how many metres from the central point the starting point of each transect would be situated. The second number specified the angle (degrees), defining the direction relative to the central point for which the start point of the transect should be placed. Combined, these two random numbers created a bearing, from the centre of the study region, that defined the transect location. The final number would randomly select between 0, 45, 90, and 180 degrees to specify the angle at which the transect should be drawn, 500 m to each side of the start point. Transects were not allowed to cross. Our measures of landscape complexity and land‐use intensity were calculated for each transect, extracted directly from Google Earth and input to our data base and averaged to give mean values of each metric for each study or substudy region. The transects sampled were line transects with no surrounding buffer.

In some cases, several studies had been conducted in the same area, in which case the same landscape data were used. When publications that garnered multiple observations had been conducted in multiple regions, and data specified per region, we collected landscape data per region. If the study region was not specified at all – but only the country – we used the mean values of all other studies in that country. The Google Maps analysed were always the most recent images available. This represents one caveat in our landscape analysis: for older studies, there is a time lag between the date the study was conducted and the date our landscape data were collected. Many of the early studies were conducted in Europe, a region that we would expect to show the least landscape change (in agricultural areas) over the relevant time span.

### Statistical Analysis

Our effect size is the log response ratio, which quantifies the proportional difference between mean species richness in conventional and organic farming (Hedges, Gurevitch & Curtis 1999). On the log scale, an effect size of 0 means no difference and a positive value means that the organic farm has higher species richness than the conventional farm. The log response ratio displays bias at small sample sizes, when the normal approximation to the distribution of the effect size deviates from the exact distribution. To assess the appropriateness of this approximation, √*n*∙μ/σ for both mean values within each effect size should be generally >3 (Hedges, Gurevitch & Curtis 1999). In our data set, only 10% of effect sizes fall below 3, while ~71% of scores exceed 6, and hence, the log response ratio is appropriate.

Our analysis was carried out using r 3.0.1 (R Development Core Team 2013) with the r package *metahdep* (Stevens & Taylor 2009). The models were fitted to the data using the function *metahdep.HBLM*. We analysed 184 separate observations for subgroups within studies – that is, different taxonomic groups or crop types. A random effect was used to account for differences across studies, for example, among farming systems included within organic and conventional groups. A grand mean effect size, across subgroups, was calculated using an intercept model (Borenstein *et al*. 2009). Variables of interest, selected *a priori*, were included in a metaregression to see whether they explained any differences in biodiversity on organic vs. conventional farms. These variables were functional groups, taxonomic groups, the three landscape measures (see Land‐Use Intensity Metrics), crop types and scale of sampling (plot, field, or farm). Uncertainty in the regression coefficients was quantified using 95% credible intervals. Credible intervals were calculated by multiplying the posterior standard error of the coefficients by the 95% point of a *t*‐distribution with *N*–*p* degrees of freedom. We estimated heterogeneity between effect sizes, τ^2^. This estimates the proportion of between‐studies variance that is true variance, as opposed to within‐study sampling error. This heterogeneity measure was used to estimate *I* ^*2*^, the proportion of total variance that is due to true heterogeneity among effect sizes (Higgins & Thompson 2002).

There is hierarchical dependence between multiple observations within studies. Having several effect sizes obtained from the same publication violates the assumption that effect sizes are independent. A publication‐level random effect allowed us to account for the dependency of multiple within‐study observations. The non‐independence among effect sizes gathered from the same publication was defined by specifying a covariance structure in the study‐specific random deviations, as parameterized by τ^2^ (Stevens & Taylor 2009). Defining dependence groups meant that a large group of within‐study effect sizes with extreme effect sizes was down‐weighted, preventing them from having a dominant effect on the overall result. By incorporating this hierarchical variance structure, we could disentangle important differences between organisms and crop types without assuming independence of observations.

The potential for bias in published results in the literature to skew synthesized results is seen as a common limitation for meta‐analyses (Borenstein *et al*. 2009; Gillman & Wright 2010). There are two ways that bias could be introduced: (i) a tendency for only ‘significantly positive’ results to be published – the ‘file drawer problem’ or (ii) studies are not representative of the population – that is, there are evidence gaps in the literature, where the question has not been investigated in certain contexts. A simple ecological example would be a lack of studies representing a system relative to its global importance; this is a bias produced by consensus in the literature that is not founded on a representative sample of reality. Meta‐analysis can provide a general quantitative synthesis. It should also describe bias in the literature and indicate where that bias may lie. We investigated bias in both the forms described above.

To investigate bias in the ‘file drawer’ context, we characterized funnel plot asymmetry in the data. The funnel plot is based on the assumption that studies with smaller sample sizes (and hence higher sampling variance) are more likely to be skewed, because they have lower statistical power; hence, negative and low‐effect results from small‐sample studies are missing from the literature. To produce a funnel plot from our hierarchical model, we plotted the residuals against precision (inverse sampling standard error; Nakagawa & Santos 2012). In combination with this funnel plot, we conducted a trim and fill assessment, whereby it is assumed that skew is due to publication bias and compensates for this by ‘filling in’ new effect sizes until the skew in the residuals is corrected for. To investigate this further, we conducted a cumulative meta‐analysis – in which studies are progressively added to the data set in the order of increasing sampling variance – which qualitatively shows how quickly the overall mean stabilizes and whether the final estimate is strongly affected by the less reliable studies. We also estimated the slope of the relationship between sampling variance and effect size. Combining these diagnostics allowed us to explore asymmetry in the data and then, under the assumption that this is due to publication bias, assess its impact on our result. The cumulative meta‐analysis approach was used to assess change in the overall effect size over time by progressively adding studies in order of publication year, and again by estimating the slope of the relationship between publication year and effect size. To investigate ‘evidence gap’ bias, we compared our data set with global data on the area of organic farming across the globe and for different crop types, collected from the FAO website [Food & Agriculture Organization of the United Nations (FAOSTAT) 2013]. We used this comparison to discuss how representative the current literature is of global organic farming trends.

## Results

The overall mean log response ratio was 0·296 (95% CI: 0·231–0·361); this indicates that species richness on organic farms is on average 34% (95% CI: 26–43) higher than conventional. The estimated standard deviation of the true effect sizes, τ, was 0·304 (variance for τ = 0·0004). This true variance among effect sizes comprised an overwhelming proportion of total variance (*I* ^*2*^ = 97·4%). These results reveal substantial heterogeneity among effect sizes, although many studies showed a large positive effect of organic farming on biodiversity relative to conventional farming. The estimate for hierarchical dependence was positive, meaning that the covariance among within‐publication effect sizes will downweight large groups of effect sizes that would otherwise have an excessive effect on the overall result.

**Figure 1:**
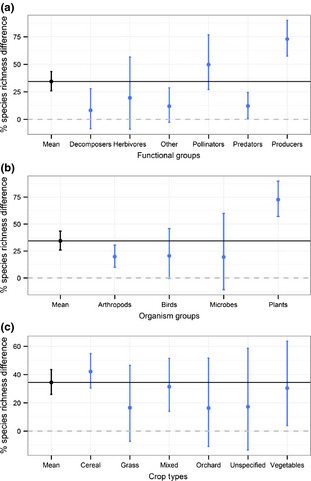
The difference in species richness (%) on organic farms, relative to conventional, classified: (a) by functional group (*n*: decomposers = 19, herbivores = 6, other = 27, pollinators = 21, predators = 49, producers = 62), (b) by organism group (*n*: arthropods = 89, birds = 17, microbes = 6, plants = 62) and (c) by crop types (*n*: cereals = 100, grasses = 13, mixed = 40, orchard = 9, unspecified = 6, vegetables = 16). The grand mean is shown in black, accompanied by the black line. The dashed lines show the zero line. 95% credible intervals are calculated from posterior standard errors.

We found large differences in the effect of organic farming on different taxonomic and functional groups (Fig. 1a,b; Table S2, Supporting information). For example, among taxonomic groups, plants benefited the most from organic farming (Fig. 1b). Arthropods, birds and microbes also showed a substantial positive effect. Disaggregating organisms into functional groups showed a variety of responses: among functional groups, the largest effect size was found for pollinators while decomposers showed little effect (Fig. 1a). The crop types showed varying responses, with large positive effect sizes in cereals and mixed farming, and moderate positive effect sizes for all others (Fig. 1c).

**Figure 2:**
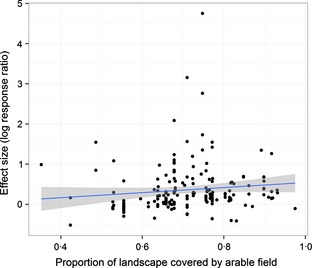
The relationship between the effect size and the proportion of the landscape covered by arable fields showing a regression slope with 95% confidence intervals.

The *percentage arable fields* had a positive effect on the magnitude of the effect size (*slope log*(*RR*) = 0·442, 95% CI: −0·089 to 0·973; Fig. 2). To assess the sensitivity of this slope estimate to the largest (‘outlying’) effect sizes, we removed the four data points with *log(RR)* >2 and reperformed the analysis; there was a small reduction in the slope estimate (0·396). Other landscape metrics had slope estimates close to zero (*number of habitats*:* log*(*RR*)* *= 0·006, 95% CI: −0·019 to 0·031; *average field size*:* log(RR)* = 0·001, 95% CI: −0·001 to 0·002). When the percentage of arable fields was fitted as an interaction with functional group, there was substantial heterogeneity in the resulting slopes. However, there was significant uncertainty in these estimates, possibly due to small sample sizes within some functional groups; thus, we choose to report this result qualitatively: increasing landscape intensity affected the magnitude of the effect size in the order: herbivores > ‘other’ > predators > producers > decomposers > pollinators. The sampling scale of species richness observation did not appreciably change the effect size (*farm* = 0·249, 95% CI: 0·161 – 0·338; treatment contrasts with *farm* scale: *field* = 0·139, 95% CI: *−*0·002 to 0·279; *plot* = −0·017, 95% CI: −0·222 to 0·187).

**Figure 3:**
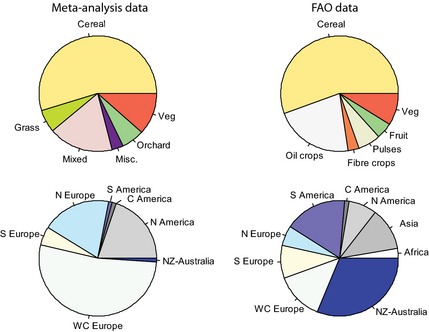
*Top row*: proportions of different crop types present in the meta‐analysis data set compared with the frequency of the most commonly grown organic crops world‐wide*. Bottom row*: geographical origin of studies in the meta‐analysis data set compared with the area under organic production in different regions of the world. FAO data obtained from their website (FAOSTAT 2013).

The representation of different crop types in the meta‐analysis was comparable with the global FAO statistics; there were similar proportions of cereals, vegetables and orchards (fruit; Fig. 3a), although fibre and oil crops were underrepresented. The geographical representation in our data set, however, showed much less congruence (Fig. 3b): Western and Northern Europe, and to some degree North America, were highly overrepresented, while studies were largely lacking from most other geographical regions, especially Asia, Africa and Australia.

**Figure 4:**
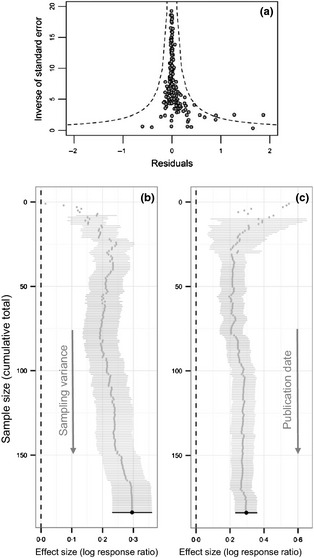
(a) Funnel plot showing asymmetry in the spread of residuals around the mean, created using the r package *meta* (Schwarzer 2010). The dashed line shows 95% confidence limits. (b) Cumulative meta‐analysis forest plot of data sorted by increasing sampling variance. (c) Cumulative meta‐analysis forest plot of data sorted by increasing publication date.

The funnel plot (Fig. 4a) showed some positive bias. A trim and fill assessment of how publication bias could impact our inference, after correcting for positive funnel plot skew, produced a negligible reduction in the effect size (−0·0001, three studies added). This suggests that, if publication bias is evident, the reported effect size is robust to its impact. Investigating further, the cumulative meta‐analysis of effect sizes sorted by sampling variance showed that less reliable studies caused the grand mean to increase, but not drastically so (Fig. 4b). If we assume that this was due to publication bias then the most conservative effect size estimate is 0·190 (95% CI: 0·135–0·246), which still corresponds to a >20% increased species richness on organic farms. This was the minimum value obtained from the cumulative plot and was reached after *c*. 80 observations (out of 184) were included. This reduced effect size did not greatly alter our interpretation of the magnitude of organic farming's positive effect on biodiversity. The relationship between sampling variance and the effect size had a positive slope (0·022, 95% CI: −0·056 to 0·101), which confirms the positive association seen in Fig. 4.

The cumulative meta‐analysis plot for data sorted by publication date (Fig. 4c) showed that the grand mean effect size estimated from our model was robust over time, although, interestingly, many of the earliest studies reported very high effect sizes. The lack of change with time was supported by a slope estimate close to zero (0·003, 95% CI: −0·007 to 0·013).

## Discussion

Our updated meta‐analysis shows that organic farming on average increases biodiversity (measured as species richness) by about one‐third relative to conventional farming. This result has been robust over the last 30 years of published studies and shows no sign of diminishing. Organic farming is therefore a tried and tested method for increasing biodiversity on farmlands and may help to reverse the continued declines of formerly common species in developed nations (Burns *et al*. 2013). Similar results have been previously obtained (Bengtsson, Ahnström & Weibull 2005; Fuller *et al*. 2005; Hole *et al*. 2005; Batáry *et al*. 2011; Garratt, Wright & Leather 2011), but our study is the most up to date, deals with the hierarchical structure of multiple within‐publication effect sizes and includes standardized measures of land‐use intensity and heterogeneity across all studies.

In other areas of biology and medicine, it has been noted that, with the addition of further evidence, effect sizes concerning a particular question often decrease over time (Jennions & Møller 2002). This is thought to occur because of initial publication bias against non‐significant or negative results that is eventually corrected. The effect size in our new study is slightly lower than the one reported in Bengtsson, Ahnström and Weibull (2005); however, our analysis reveals that the grand mean effect size is robust over time (Fig. 4c). There is therefore no sign of a dwindling effect size with the addition of further evidence. This implies that the increase in diversity with organic farming that we report here is robust, given the choice of crops and study areas included (see below for a discussion of the representativeness of our study).

### Land‐Use Intensity Effects

Many authors have speculated on and investigated the importance of landscape characteristics in shaping the likely effect of organic farming on biodiversity (Bengtsson, Ahnström & Weibull 2005; Rundlöf & Smith 2006; Rundlöf, Bengtsson & Smith 2008; Rundlöf, Nilsson & Smith 2008; Batáry *et al*. 2011). Here, we calculated three standardized measures of land‐use intensity and heterogeneity for all studies: the proportion of arable fields, the typical field size and the number of habitats. Only the proportion of arable fields in the landscape had any significant overall effect. The difference in diversity between organic and conventional farming generally increased with increasing proportion of arable fields, although there was large variation around the estimated slope. Some of this variance may be due to different responses between functional groups (Batáry *et al*. 2011). The slope of this relationship decreased in the order: decomposers > ‘other’ > predators > herbivores > producers > pollinators, suggesting that the effect of organic farming on predators is greater in intensively managed landscapes, whereas the effect of organic farming on pollinators does not increase much with land‐use intensity. These differences may be due to the importance of local actions relative to regional actions and to the movement of organisms and chemicals across the landscape. For example, some pollinators are known to be sensitive to certain pesticides (Goulson 2013), leading to an EU moratorium on neonicotinoids. If an organic farmer refrains from using pesticides, then local pollinator richness might increase; however, given that these chemicals might drift substantially, and that pollinators on an organic farm will likely visit neighbouring farms, the impact of this local action might have no more effect in an intensively managed landscape compared with an extensive one.

### Organism Groups, Crop Types and Spatial Scale

We expected that the magnitude of the positive effect of organic farming would vary among organism groups, as this has been found repeatedly (Bengtsson, Ahnström & Weibull 2005; Fuller *et al*. 2005; Batáry *et al*. 2011; Garratt, Wright & Leather 2011; Winqvist *et al*. 2011; Winqvist, Ahnström & Bengtsson 2012). As in previous studies, we found that plants benefited most from organic farming, probably because of restricted herbicide use (Roschewitz *et al*. 2005; Rundlöf, Edlund & Smith 2010). Arthropods, birds and microbes also benefited, with varying levels of estimated confidence. Accordingly, most functional groups – herbivores, pollinators, predators and producers – were more diverse in organic farming, with the exception of decomposers. The lack of positive effects on decomposers, which are mostly soil fauna, is surprising given that there are positive effects of organic farming on soil conditions and soil carbon (Mäder *et al*. 2002; Gattinger *et al*. 2012). This may be because variation in soil type and structure is more important for soil organisms than the farming system itself. Such interactions between factors influencing the diversity and abundance of soil organisms would repay more investigation. The strong positive effects of organic farming on herbivores and pollinators are consistent with other studies (Rundlöf & Smith 2006; Holzschuh, Steffan‐Dewenter & Tscharntke 2008; Rundlöf, Bengtsson & Smith 2008; Garratt, Wright & Leather 2011).

We found significant differences in the effect of organic farming among crop types. In cereal fields, which comprised >50% of the studies, organic farming had large effects, significantly higher than in vegetable crops and orchards (Fig. 1c). This might reflect the intensive management of conventional cereal crops, with repeated applications of inorganic fertilizers and fungicides. The effect size in both vegetable crops and orchards, although positive, did not differ significantly from zero, but this could be due to small sample sizes. A lower but still significant effect was found in grasslands (pastures and permanent or semi‐permanent leys), which are generally not so intensively managed. The number of studies in grasslands, vegetables and orchards was quite low, and we recommend that these crops are given more attention in the future.

In a previous meta‐analysis (Bengtsson, Ahnström & Weibull 2005), small‐scale studies (on the plot or single field scale) showed much larger effect sizes than studies on larger spatial scales. However, we found negligible differences across scales. This suggests that the general benefit of organic farming is robust across sampling scales, in contrast to recent work that suggests that this benefit diminishes at larger scales (Gabriel *et al*. 2010; Crowder *et al*. 2012). The previous meta‐analysis result may have been due to small sample size or publication bias, which highlights the importance of updating meta‐analyses with additional evidence. We note that most of the recent studies have been conducted at the farm scale, which is the most relevant scale for evaluating both organic farming as an agrienvironmental scheme for biodiversity, and for the sustainability of farming systems in general.

### Publication Bias

The funnel plot suggests a positively biased spread of effect sizes (Fig. 4a), which could be interpreted as a tendency for studies showing large positive effects of conventional farming on biodiversity to remain unpublished. However, an alternative interpretation may be that large positive effects of organic farming occur occasionally, while large positive effects of conventional farming are exceptionally unlikely. This seems reasonable given the nonlinear nature of many natural processes, for example population growth, which could occasionally fuel very large impacts of not controlling certain groups of organisms. In any case, the positive bias is slight and has been shown to not affect our result.

Previous studies of organic farming on biodiversity have been strongly biased towards temperate Western and Northern Europe and North America (Fig. 3), that is, intensive farming systems in developed countries. There is extremely limited data available from other areas of the world, for example, Eastern Europe, Asia, Africa, Central and Southern America, a bias also noted by Batáry *et al*. (2011), Martin, Blossey and Ellis (2012), and Randall and James (2012). We therefore recommend that studies of organic farming practices on diversity in tropical and subtropical areas (e.g. Deb 2009; Zhang *et al*. 2013) should receive high priority. It is, for example, surprising that there are no studies on organic bananas or cacao, despite these products being widely available in European supermarkets. Mediterranean climates are also underrepresented, although a few studies from California (Drinkwater *et al*. 1995; Letourneau & Bothwell 2008; Kremen, Iles & Bacon 2012) and South Africa (Kehinde & Samways 2012) exist.

### The Organic Controversy

The yields from organic farms are generally lower than conventional yields, although some controversy exists concerning the size of this effect and whether it is more prominent in developed countries (Badgley *et al*. 2007; De Ponti, Rijk & van Ittersum 2012; Dobermann 2012; Reganold 2012; Seufert, Ramankutty & Foley 2012). As outlined in the introduction, this implies a potential trade‐off between biodiversity and crop yields. For example, Gabriel *et al*. (2013) in a study of cereal crops in Southern England concluded that the benefits of organic farming to biodiversity were entirely bought at the cost of reduced yield. They further suggested that the lower yields of organic farming may therefore have the unfortunate result of increasing the total area of land under agricultural production. However, there are other, often unmeasured, potential positive environmental benefits of organic farming. For example, nitrogen and phosphorus pollution caused by leaching from intensively managed fields is still a major problem in many countries and incurs significant costs to society (Heathwaite, Sharpley & Gburek 2000). An overall evaluation of organic farming in relation to crop yields therefore needs to account for the effects of farming practice on a wider range of environmental factors (Mondelaers, Aertsens & Huylenbroeck 2009; Sandhu, Wratten & Cullen 2010; Gattinger *et al*. 2012; Bommarco, Kleijn & Potts 2013).

### Synthesis and Recommendations

This analysis affirms that organic farming usually has large positive effects on average species richness compared with conventional farming. Given the large areas of land currently under agricultural production, organic methods could undoubtedly play a major role in halting the continued loss of diversity from industrialized nations. The effect of organic farming varied with the organism group and crop studied, and with the proportion of arable land in the surrounding landscape. We found larger effects in cereals, among plants and pollinators, and in landscapes with higher land‐use intensity. Despite the fact that organic farming has been suggested to have large effects on soil conditions, its effects on soil organisms were ambiguous and in general understudied. Finally, it is clear that three decades of studying the effects of organic farming on biodiversity have been heavily biased towards agricultural systems in the developed world, especially Europe and North America. We therefore recommend that other regions and agricultural systems are given much greater attention. In particular, more studies are needed in tropical, subtropical and Mediterranean climates. Studies at any scale would be beneficial: at the farm scale because this is the economic unit of farming, and at the landscape scale because this is the scale at which many organisms respond. This would allow a more balanced and globally relevant assessment of organic farming effects on biodiversity, ecosystem services, food production and agricultural sustainability.

## Author contributions

SLT, JB and LAT designed the analyses and drafted the paper; SLT performed the analyses; JA, CW and FM collected the data. The original idea for the study emerged from discussions between JB, LAT, JA, FM and CW.

## Data accessibility

Meta‐analysis data and R script: DRYAD entry doi: 10·5061/dryad.609t7 (Tuck *et al*. 2014). FAO statistics on organic farming coverage: FAOSTAT (2013).

## Supporting information


**Appendix S1.** List of studies included in the meta‐analysis.

**Appendix S2.** prisma flowchart showing the data collection decision process.

**Table S1.** Model estimates.

**Table S2.** Coefficient estimates for subgroups included in Fig. 1.

**Table S3.** List of studies rejected during data collection, with reasons for rejection.
