# Supplementary material for: Land‐use intensity and the effects of organic farming on biodiversity: a hierarchical meta‐analysis
Source: J Appl Ecol. 2014 Feb 7;51(3):746–55. doi: 10.1111/1365-2664.12219 (PMC4299503; doi:10.1111/1365-2664.12219)
Supplement: Supplementary file 1 — Appendix S1. List of studies included in the meta‐analysis. [file JPE-51-746-s001.doc]

**Appendix S1**. Bibliography for studies included in the meta-analysis.

Aavik, T. & Liira, J. (2010) Quantifying the effect of organic farming, field boundary type and landscape structure on the vegetation of field boundaries. *Agriculture, ecosystems & environment*, **135**, 178–186.

Armstrong, G. (1995) Carabid beetle (Coleoptera: Carabidae) diversity and abundance in organic potatoes and conventionally grown seed potatoes in the north of Scotland. *Pedobiologia*, **39**, 231–237.

Aude, E., Tybirk, K. & Pedersen, M.B. (2003) Vegetation diversity of conventional and organic hedgerows in Denmark. *Agriculture Ecosystems & Environment*, **99**, 135–147.

Bithell, S.L., Booth, L.H., Wratten, S.D. & Heppelthwaite, V.J. (2005) Earthworm populations and association with soil parameters in organic and conventional ley pastures. *Biological Agriculture & Horticulture*, **23**, 143–159.

Boutin, C., Baril, A. & Martin, P.A. (2008) Plant diversity in crop fields and woody hedgerows of organic and conventional farms in contrasting landscapes. *Agriculture Ecosystems & Environment*, **123**, 185–193.

Boutin, C., Martin, P.A. & Baril, A. (2009) Arthropod diversity as affected by agricultural management (organic and conventional farming), plant species, and landscape context. *Ecoscience*, **16**, 492–501.

Boutin, C., Baril, A., McCabe, S.K., Martin, P.A. & Guy, M. (2011) The Value of Woody Hedgerows for Moth Diversity on Organic and Conventional Farms. *Environmental Entomology*, **40**, 560–569.

Brittain, C., Bommarco, R., Vighi, M., Settele, J. & Potts, S.G. (2010) Organic farming in isolated landscapes does not benefit flower-visiting insects and pollination. *Biological Conservation*, **143**, 1860–1867.

Bruggisser, O.T., Schmidt-Entling, M.H. & Bacher, S. (2010) Effects of vineyard management on biodiversity at three trophic levels. *Biological Conservation*, **143**, 1521–1528.

BTO. (1995) *The Effect of Organic Farming Regimes on Breeding and Winter Bird Populations*. Research report No.154. British Trust for Ornithology: Norfolk, UK.

Caballero-Lopez, B., Blanco-Moreno, J.M., Perez, N., Pujade-Villar, J., Ventura, D., Oliva, F. & Sans, F.X. (2010) A functional approach to assessing plant-arthropod interaction in winter wheat. *Agriculture, ecosystems & environment*, **137**, 288–293.

Christensen, K.D., Jacobsen, E.M. & Nøhr, H. (1996) A comparative study of bird faunas in conventionally and organically farmed areas. *Dansk Ornithologisk Forenings Tidskrift*, **90**, 21–28.

Clough, Y., Kruess, A. & Tscharntke, T. (2007) Local and landscape factors in differently managed arable fields affect the insect herbivore community of a non-crop plant species. *Journal of Applied Ecology*, **44**, 22–28.

Clough, Y., Kruess, A. & Tscharntke, T. (2007) Organic versus conventional arable farming systems: Functional grouping helps understand staphylinid response. *Agriculture Ecosystems & Environment*, **118**, 285–290.

Clough, Y., Kruess, A., Kleijn, D. & Tscharntke, T. (2005) Spider diversity in cereal fields: comparing factors at local, landscape and regional scales. *Journal of Biogeography*, **32**, 2007–2014.

Crowder, D.W., Northfield, T.D., Strand, M.R. & Snyder, W.E. (2010) Organic agriculture promotes evenness and natural pest control. *Nature*, **466**, 109–112.

Dänhardt, J., Green, M., Lindström, Å., Rundlöf, M. & Smith, H.G. (2010) Farmland as stopover habitat for migrating birds – effects of organic farming and landscape structure. *Oikos*, **119**, 1114–1125.

Diekötter, T., Wamser, S., Wolters, V. & Birkhofer, K. (2010) Landscape and management effects on structure and function of soil arthropod communities in winter wheat. *Agriculture, Ecosystems & Environment*, **137**, 108–112.

Doles J.L., Zimmerman R.J. & Moore J.C. (2001) Soil microarthropod community structure and dynamics in organic and conventionally managed apple orchards in Western Colorado, USA. *Applied Soil Ecology*, **18**, 83–96.

Döring, T.F., Hiller, A., Wehke, S., Schulte, G. & Broll, G. (2003) Biotic indicators of carabid species richness on organically and conventionally managed arable fields. *Agriculture, Ecosystems & Environment*, **98**, 133–139.

Drinkwater, L.E., Letourneau, D.K., Workneh, F., van Bruggen, A.H.C. & Shennan, C. (1995) Fundamental Differences Between Conventional and Organic Tomato Agroecosystems in California. *Ecological Applications*, **5**, 1098.

Dritschilo, W. & Erwin, T.L. (1982) Responses in Abundance and Diversity of Cornfield Carabid Communities to Differences in Farm Practices. *Ecology*, **63**, 900.

Dritschilo, W. & Wanner, D. (1980) Ground Beetle Abundance In Organic and Conventional Corn Fields. *Environmental Entomology*, **9**, 629–631.

Ekroos, J., Heliölä, J. & Kuussaari, M. (2010) Homogenization of lepidopteran communities in intensively cultivated agricultural landscapes. *Journal of Applied Ecology*, **47**, 459–467.

Ekroos, J., Piha, M. & Tiainen, J. (2008) Role of organic and conventional field boundaries on boreal bumblebees and butterflies. *Agriculture Ecosystems & Environment*, **124**, 155–159.

Ekroos, J., Hyvönen, T., Tiainen, J. & Tiira, M. (2010) Responses in plant and carabid communities to farming practises in boreal landscapes. *Agriculture, Ecosystems & Environment*, **135**, 288–293.

Feber, R.E., Johnson, P.J., Firbank, L.G., Hopkins, A. & Macdonald, D.W. (2007) A comparison of butterfly populations on organically and conventionally managed farmland. *Journal of Zoology*, **273**, 30–39.

Feber, R.E., Bell, J., Johnson, P.J., Firbank, L.G. & MacDonald, D.W. (1998) The Effects of Organic Farming on Surface-Active Spider (Araneae) Assemblages in Wheat in Southern England, UK. *Journal of Arachnology*, **26**, 190–202.

Fischer, C., Thies, C. & Tscharntke, T. (2011) Small mammals in agricultural landscapes: Opposing responses to farming practices and landscape complexity. *Biological conservation*, **144**, 1130–1136.

Fischer, C., Flohre, A., Clement, L.W., Batary, P., Weisser, W.W., Tscharntke, T. & Thies, C. (2011) Mixed effects of landscape structure and farming practice on bird diversity. *Agriculture, ecosystems & environment*, **141**, 119–125.

Flohre, A., Rudnick, M., Traser, G., Tscharntke, T. & Eggers, T. (2011) Does soil biota benefit from organic farming in complex vs. simple landscapes? Agriculture, Ecosystems & Environment, 141, 210-214.

Fluetsch, K.M. & Sparling, D.W. (1994) Avian nesting success and diversity in conventionally and organically managed apple orchards. *Environmental Toxicology and Chemistry*, **13**, 1651–1659.

Foissner W. (1997) Protozoa as bioindicators in agroecosystems, with emphasis on farming practices, biocides, and biodiversity. Agriculture, Ecosystems and Environment, 62, 93–103.

Gabriel, D. & Tscharntke, T. (2007) Insect pollinated plants benefit from organic farming. *Agriculture Ecosystems & Environment*, **118**, 43–48.

Gabriel, D., Sait, S.M., Hodgson, J.A., Schmutz, U., Kunin, W.E. & Benton, T.G. (2010) Scale matters: the impact of organic farming on biodiversity at different spatial scales. *Ecology Letters*, **13**, 858–869.

Galván, G.A., Parádi, I., Burger, K., Baar, J., Kuyper, T.W., Scholten, O.E. & Kik, C. (2009) Molecular diversity of arbuscular mycorrhizal fungi in onion roots from organic and conventional farming systems in the Netherlands. *Mycorrhiza*, **19**, 317–328.

Genghini, M., Gellini, S. & Gustin, M. (2006) Organic and integrated agriculture: the effects on bird communities in orchard farms in northern Italy. *Biodiversity and Conservation*, **15**, 3077–3094.

Granqvist, Å. (1999) *Mångfalden Av Växter i Jordbrukslandskapet – Effekten Av Odlingssystem Och Landskapsheterogenitet. Examensarbeten/Seminarieuppsatser 4. (Diversity of Plants in the Agricultural Landscape. BSc Report)*. Department of Ecology and Crop Production Science, SLU. Uppsala. (also reported in Weibull *et al*. 2002) (In Swedish).

Hawes, C., Squire, G.R., Hallett, P.D., Watson, C.A. & Young, M. (2010) Arable plant communities as indicators of farming practice. *Agriculture, ecosystems & environment*, **138**, 17–26.

Hesler, L.S., Grigarick, A.A., Oraze, M.J. & Palrang, A.T. (1993) Arthropod Fauna of Conventional and Organic Rice Fields in California. *Journal of Economic Entomology*, **86**, 149–158.

Hokkanen, H. & Holopainen, J.K. (1986) Carabid species and activity densities in biologically and conventionally managed cabbage fields. *Journal of Applied Entomology*, **102**, 353–363.

Holzschuh, A., Steffan-Dewenter, I., Kleijn, D. & Tscharntke, T. (2007) Diversity of flower-visiting bees in cereal fields: effects of farming system, landscape composition and regional context. *Journal of Applied Ecology*, **44**, 41–49.

Holzschuh, A., Steffan-Dewenter, I. & Tscharntke, T. (2008) Agricultural landscapes with organic crops support higher pollinator diversity. *Oikos*, **117**, 354–361.

Holzschuh, A., Steffan-Dewenter, I. & Tscharntke, T. (2010) How do landscape composition and configuration, organic farming and fallow strips affect the diversity of bees, wasps and their parasitoids? *Journal of Animal Ecology*, **79**, 491–500.

Hutton, S.A. & Giller, P.S. (2003) The effects of the intensification of agriculture on northern temperate dung beetle communities. *Journal of Applied Ecology*, **40**, 994–1007.

Hyvonen, T., Ketoja, E., Salonen, J., Jalli, H. & Tiainen, J. (2003) Weed species diversity and community composition in organic and conventional cropping of spring cereals. *Agriculture Ecosystems & Environment*, **97**, 131–149.

Irmler, U. (2003) The spatial and temporal pattern of carabid beetles on arable fields in northern Germany (Schleswig-Holstein) and their value as ecological indicators. *Agriculture, Ecosystems & Environment*, **98**, 141–151.

Jonason, D., Andersson, G.K.S., Öckinger, E., Rundlöf, M., Smith, H.G. & Bengtsson, J. (2011) Assessing the effect of the time since transition to organic farming on plants and butterflies. *Journal of Applied Ecology*, **48**, 543–550.

Jose-Maria L.; Sans F. X. (2011) Weed seedbanks in arable fields: effects of management practices and surrounding landscape. *Weed Research*, 51, 631-640.

Krauss, J., Gallenberger, I. & Steffan-Dewenter, I. (2011) Decreased Functional Diversity and Biological Pest Control in Conventional Compared to Organic Crop Fields. *PLoS ONE*, **6**, e19502.

Kremen, C., Williams, N.M. & Thorp, R.W. (2002) Crop pollination from native bees at risk from agricultural intensification. *Proceedings of the National Academy of Sciences*, **99**, 16812–16816.

Kromp, B. (1989) Carabid beetle communities (Carabidae, Coleoptera) in biologically and conventionally farmed agroecosystems. *Agriculture, ecosystems & environment*, **27**, 241–251.

Kromp, B. (1990) Carabid beetles (Coleoptera, Carabidae) as bioindicators in biological and conventional farming in Austrian potato fields. *Biology and Fertility of Soils*, **9**, 182–187.

Letourneau, D.K. & Bothwell, S.G. (2008) Comparison of organic and conventional farms: challenging ecologists to make biodiversity functional. *Frontiers in Ecology and the Environment*, **6**, 430–438.

Lokemoen, J.T. & Beiser, J.A. (1997) Bird Use and Nesting in Conventional, Minimum-Tillage, and Organic Cropland. *The Journal of Wildlife Management*, **61**, 644–655.

Macfadyen, S., Gibson, R., Polaszek, A., Morris, R.J., Craze, P.G., Planque, R., Symondson, W.O.C. & Memmott, J. (2009) Do differences in food web structure between organic and conventional farms affect the ecosystem service of pest control? *Ecology Letters*, **12**, 229–238.

Mäder, P., Fliessbach, A., Dubois, D., Gunst, L., Fried, P. & Niggli, U. (2002) Soil Fertility and Biodiversity in Organic Farming. *Science*, **296**, 1694–1697.

Manhoudt, A.G.E., Visser, A.J. & de Snoo, G.R. (2007) Management regimes and farming practices enhancing plant species richness on ditch banks. *Agriculture Ecosystems & Environment*, **119**, 353–358.

Martínez-Sánchez, J.C. (2008) *The Role of Organic Production in Biodiversity Conservation in Shade Coffee Plantations*. PhD Thesis, Department of Biology, University of Washington, USA.

Menalled, F.D., Gross, K.L. & Hammond, M. (2001) Weed aboveground and seedbank community responses to agricultural management systems. *Ecological Applications*, **11**, 1586–1601.

Moreby, S.J., Aebischer, N.J., Southway, S.E. & Sotherton, N.W. (1994) A comparison of the flora and arthropod fauna of organically and conventionally grown winter wheat in southern England. *Annals of Applied Biology*, **125**, 13–27.

Oberg, S. (2007) Diversity of spiders after spring sowing – influence of farming system and habitat type. *Journal of Applied Entomology*, **131**, 524–531.

Pelosi, C., Bertrand, M. & Roger-Estrade, J. (2009) Earthworm community in conventional, organic and direct seeding with living mulch cropping systems. *Agronomy for Sustainable Development*, **29**, 287–295.

Petersen, S., Axelsen, J.A., Tybirk, K., Aude, E. & Vestergaard, P. (2006) Effects of organic farming on field boundary vegetation in Denmark. *Agriculture Ecosystems & Environment*, **113**, 302–306.

Pfiffner, L. & Mäder, L. (1997) Effects of biodynamic, organic and conventional production systems on earthworm populations. *Biological Agriculture & Horticulture*, **15**, 3–10.

Pfiffner, L. & Niggli, U. (1996) Effects of bio-dynamic, organic and conventional farming on ground beetles (Col. Carabidae) and other epigaeic arthropods in winter wheat. *Biological Agriculture & Horticulture*, **12**, 353–364.

Pollnac, F.W., Maxwell, B.D. & Menalled, F.D. (2009) Using Species-Area Curves to Examine Weed Communities in Organic and Conventional Spring Wheat Systems. *Weed Science*, 57(3), 241-247.

Ponce, C., Bravo, C., Garcia De Leon, D., Magana, M. & Alonso, J.C. (2011) Effects of organic farming on plant and arthropod communities: A case study in Mediterranean dryland cereal. *Agriculture, ecosystems & environment*, **141**, 193–201.

Power, E.F. & Stout, J.C. (2011) Organic dairy farming: impacts on insect–flower interaction networks and pollination. *Journal of Applied Ecology*, **48**, 561–569.

Purtauf, T., Roschewitz, I., Dauber, J., Thies, C., Tscharntke, T. & Wolters, V. (2005) Landscape context of organic and conventional farms: Influences on carabid beetle diversity. *Agriculture Ecosystems & Environment*, **108**, 165–174.

Reddersen, J. (1997) The Arthropod Fauna of Organic Versus Conventional Cereal Fields in Denmark. *Biological Agriculture & Horticulture*, **15**, 61–71.

Rundlöf, M. & Smith, H.G. (2006) The effect of organic farming on butterfly diversity depends on landscape context. *Journal of Applied Ecology*, **43**, 1121–1127.

Rundlöf, M., Nilsson, H. & Smith, H.G. (2008) Interacting effects of farming practice and landscape context on bumblebees. *Biological Conservation*, **141**, 417–426.

Rundlöf, M., Edlund, M. & Smith, H.G. (2010) Organic farming at local and landscape scales benefits plant diversity. *Ecography*, **33**, 514–522.

Salonen, J. & Hyvönen, T. (2011) *From Surveys to an Indicator – a Case Study with Arable Weeds*. NJF Seminar 442.Nordic Meeting on Agricultural Statistics – A joint workshop of statisticians and research community. NJF: Oslo, Norway.

Salonen, J., Hyvönen, T. & Jalli, H. (2001) Weeds in spring cereal fields in Finland – a third survey. *Agricultural and Food Science*, **10**, 347–364.

Sanchez-Moreno, S., Nicola, N.L., Ferris, H. & Zalom, F.G. (2009) Effects of agricultural management on nematode-mite assemblages: Soil food web indices as predictors of mite community composition. *Applied Soil Ecology*, **41**, 107–117.

Schmidt, M.H., Roschewitz, I., Thies, C. & Tscharntke, T. (2005) Differential effects of landscape and management on diversity and density of ground-dwelling farmland spiders. *Journal of Applied Ecology*, **42**, 281–287.

Smith, R.G & Gross, K.L. (2006) Weed community and corn yield variability in diverse managemnet systems. *Weed Science*, 54, 106-113.

Smith, H.G., Dänhardt, J., Lindström, Å. & Rundlöf, M. (2010) Consequences of organic farming and landscape heterogeneity for species richness and abundance of farmland birds. *Oecologia*, **162**, 1071–1079.

Thomas, P.J., Martin, P. & Boutin, C. (2011) Bush, bugs, and birds; interdependency in a farming landscape. *Open Journal of Ecology*, **1**, 9–23.

Tyser, L., Novakova, K., Hamouz, P. & Necasova, M. (2008) Species diversity of weed communities in conventional and organic farming systems in the Czech Republic. *Journal of Plant Diseases and Protection*, 291–295.

Ulber, L., Steinmann, H.H., Klimek, S. & Isselstein, J. (2009) An on-farm approach to investigate the impact of diversified crop rotations on weed species richness and composition in winter wheat. *Weed Research*, **49**, 534–543.

Van Diepeningen, A.D., de Vos, O.J., Korthals, G.W. & van Bruggen, A.H.C. (2006) Effects of organic versus conventional management on chemical and biological parameters in agricultural soils. *Applied Soil Ecology*, **31**, 120–135.

Van der Gast, C.J., Gosling, P., Tiwari, B. & Bending, G.D. (2011) Spatial scaling of arbuscular mycorrhizal fungal diversity is affected by farming practice. *Environmental Microbiology*, **13**, 241–249.

Verbruggen, E., Röling, W.F.M., Gamper, H.A., Kowalchuk, G.A., Verhoef, H.A. & van der Heijden, M.G.A. (2010) Positive effects of organic farming on below-ground mutualists: large-scale comparison of mycorrhizal fungal communities in agricultural soils. *New Phytologist*, **186**, 968–979.

Vesely, M. & Sarapatka, B. (2008) Effects of conversion to organic farming on carabid beetles (Carabidae) in experimental fields in the Czech Republic. *Biological Agriculture & Horticulture*, **25**, 289–309.

Weibull, A.C. & Ostman, O. (2003) Species composition in agroecosystems: The effect of landscape, habitat, and farm management. *Basic and Applied Ecology*, **4**, 349–361.

Weibull, A.-C., Bengtsson, J. & Nohlgren, E. (2000) Diversity of butterflies in the agricultural landscape: the role of farming system and landscape heterogeneity. *Ecography*, **23**, 743–750.

Winqvist, C., Bengtsson, J., Aavik, T., Berendse, F., Clement, L.W., Eggers, S., Fischer, C., Flohre, A., Geiger, F., Liira, J., Pärt, T., Thies, C., Tscharntke, T., Weisser, W.W. & Bommarco, R. (2011) Mixed effects of organic farming and landscape complexity on farmland biodiversity and biological control potential across Europe. *Journal of Applied Ecology*, **48**, 570–579.

Wortman, S., Lindquist, J., Haar, M. & Francis, C. (2010) Increased weed diversity, density and above-ground biomass in long-term organic crop rotations. *Agronomy & Horticulture – Faculty Publications*.

Yeates, G.W., Bardgett, R.D., Cook, R., Hobbs, P.J., Bowling, P.J. & Potter, J.F. (1997) Faunal and Microbial Diversity in Three Welsh Grassland Soils Under Conventional and Organic Management Regimes. *Journal of Applied Ecology*, **34**, 453–470.

Younie, D. & Armstrong, G. (1995) Botanical and invertebrate diversity in organic and intensively fertilised grassland. *Land use and biodiversity: The role of organic farming*, 35–44.

Zalazar, L. & Salvo, A. (2007) Entomofauna associated to horticultural crops under organic and conventional practices in Cordoba, Argentina. *Neotropical Entomology*, **36**, 765–773.
