# Supplementary material for: Land‐use intensity and the effects of organic farming on biodiversity: a hierarchical meta‐analysis
Source: J Appl Ecol. 2014 Feb 7;51(3):746–55. doi: 10.1111/1365-2664.12219 (PMC4299503; doi:10.1111/1365-2664.12219)
Supplement: Supplementary file 2 — Appendix S2. prisma flowchart showing the data collection decision process. [file JPE-51-746-s002.doc]

**Appendix S2**. PRISMA flow diagram representing the flow of information through the decision process (i.e., the number of studies identified, rejected, and accepted). Lists of papers that were rejected or accepted are in other appendices. This flow chart was adapted from the PRISMA template found on their website (Moher et al. 2009).

**Screening**

**Included**

**Eligibility**

**Identification**

Records identified through database searching
(n = 834)

Additional records identified through other sources
(n = 96)

Records after duplicates removed
(n = 930)

Records screened
(n = 930)

Records excluded
(n = 670)

Full-text articles assessed for eligibility
(n = 260)

Full-text articles excluded, with reasons
(n = 166)

Studies included in quantitative synthesis (meta-analysis)
(n = 94; 63 found via database searching and 31 via other sources)
