# Supplementary material for: Land‐use intensity and the effects of organic farming on biodiversity: a hierarchical meta‐analysis
Source: J Appl Ecol. 2014 Feb 7;51(3):746–55. doi: 10.1111/1365-2664.12219 (PMC4299503; doi:10.1111/1365-2664.12219)
Supplement: Supplementary file 3 — Table S1. Model estimates. [file JPE-51-746-s003.doc]

**Table S1.** The full model output (treatment contrasts). Significance levels refer to two-sided tail posterior probabilities that a parameter estimate based on replicated data will be as extreme the estimate shown. The 95% credible intervals are calculated using posterior standard errors for the coefficients, multiplied by the 95% point of a *t*-distribution with *N* - *p* degrees of freedom. All estimates are intercepts, except the landscape slope metrics. The two landscape slope metrics are centred on their means so that the subgroup contrasts shown represent the mean effect for that contrast at the mean level of the landscape metrics. Model:

~ functional.groups + crop.groups + scale.groups + c(% arable fields) + c(mean.field.size)

|  | **Effect size (log response ratio)** | **95% CI: lower** | **95% CI: upper** | **Significance** |
| --- | --- | --- | --- | --- |
| Intercept (Decomposers) | 0.097735404 | -0.12104987 | 0.31652068 | 0.3762077 |
| Herbivores | 0.069541361 | -0.26504002 | 0.40412275 | 0.6784707 |
| Pollinators | 0.322264682 | 0.07078753 | 0.57374183 | 0.01101670 |
| Predators | 0.007759126 | -0.20107883 | 0.21659708 | 0.9416302 |
| Producers | 0.461473543 | 0.25414176 | 0.66880533 | < 0.001 |
| Other | 0.033730333 | -0.20558783 | 0.27304850 | 0.7810916 |
| Grasses | -0.166191468 | -0.41059206 | 0.07820912 | 0.1788231 |
| Orchard | -0.079348634 | -0.35641011 | 0.19771284 | 0.5686324 |
| Vegetables | 0.092544056 | -0.15576454 | 0.34085265 | 0.4608277 |
| Mixed | -0.031065434 | -0.19567530 | 0.13354443 | 0.7052821 |
| Unspecified | -0.146028600 | -0.45379428 | 0.16173708 | 0.3473713 |
| Field scale | 0.046611614 | -0.09732956 | 0.19055279 | 0.5195993 |
| Plot scale | -0.040068724 | -0.24548388 | 0.16534643 | 0.6997389 |
| % landscape arable field | 0.442203792 | -0.08874636 | 0.97315394 | 0.09956164 |
| Number of habitats | 0.005879041 | -0.01893249 | 0.03069057 | 0.6406567 |
