# Supplementary material for: Land‐use intensity and the effects of organic farming on biodiversity: a hierarchical meta‐analysis
Source: J Appl Ecol. 2014 Feb 7;51(3):746–55. doi: 10.1111/1365-2664.12219 (PMC4299503; doi:10.1111/1365-2664.12219)
Supplement: Supplementary file 4 — Table S2. Coefficient estimates for subgroups included in Fig. 1. [file JPE-51-746-s004.doc]

**Table S2.** Table showing the estimates and credible intervals in the coefficient plots (Fig. 1). The sample sizes, *n*, for each subgroup are shown. Estimate of the grand mean effect size followed by the estimates for each subgroup. The estimates are transformed to the original scale (antilog of the log response ratios), then expressed as percentage species richness difference in organic farms, relative to conventional (100 * (exp(log response ratio) – 1)). Credible intervals are the posterior standard errors multiplied by the 95% point of a *t*-distribution with *N* - *p* degrees of freedom.

|  | **Effect size (% species richness difference)** | **95% CI: lower** | **95% CI: upper** | ***n*** |
| --- | --- | --- | --- | --- |
| Grand mean | 34.42387 | 25.97721 | 43.43687 | 184 |
| **Functional groups** | | | | |
| Decomposers | 8.124467 | -8.502363 | 27.77270 | 19 |
| Herbivores | 19.442734 | -8.872662 | 56.55639 | 6 |
| Pollinators | 49.770856 | 26.940170 | 76.70773 | 21 |
| Predators | 12.135662 | 1.062716 | 24.42182 | 49 |
| Producers | 72.930327 | 57.495454 | 89.87785 | 62 |
| Other | 11.855023 | -2.677181 | 28.55717 | 27 |
| **Organism groups** | | | | |
| Arthropods | 19.809328 | 10.0150127 | 30.47560 | 89 |
| Birds | 20.502156 | -0.4095779 | 45.80488 | 17 |
| Earthworms | -27.532907 | -54.1360975 | 14.50137 | 4 |
| Nematodes | 14.724595 | -23.8671371 | 72.87847 | 2 |
| Mammals | -2.200489 | -49.1196318 | 87.98497 | 1 |
| Plants | 72.583275 | 56.9755180 | 89.74288 | 62 |
| Protozoa | 1.827472 | -33.3365440 | 55.54000 | 3 |
| Microbes | 19.417791 | -10.7828830 | 59.84162 | 6 |
| **Crop groups** | | | | |
| Cereals | 42.09368 | 30.480062 | 54.74100 | 100 |
| Grasses | 16.55607 | -7.226563 | 46.43543 | 13 |
| Mixed | 31.41508 | 14.057352 | 51.41438 | 40 |
| Orchard | 16.26433 | -10.835507 | 51.60065 | 9 |
| Unspecified | 17.20750 | -13.398012 | 58.62914 | 6 |
| Vegetables | 30.34612 | 3.886768 | 63.54451 | 16 |
